# 1014. Assessment of racial, ethnic, and sex-based disparities in time-to-antibiotics and sepsis outcomes in a large multi-hospital cohort

**DOI:** 10.1093/ofid/ofad500.045

**Published:** 2023-11-27

**Authors:** Theodore R Pak, Sarimer Sanchez, Caroline S McKenna, Chanu Rhee, Michael Klompas

**Affiliations:** Massachusetts General Hospital, Boston, Massachusetts; Boston Public Health Commission, Bostom, Massachusetts; Harvard Pilgrim Health Care Institute, Boston, Massachusetts; Brigham and Women's Hospital, Boston, Massachusetts; Harvard Medical School and Harvard Pilgrim Health Care Institute, Boston, Massachusetts

## Abstract

**Background:**

Observational studies suggest that women and people of color have worse sepsis outcomes compared to White men. However, many of these studies were underpowered, adjusted for limited confounders, or did not evaluate care factors such as time-to-antibiotics.

**Methods:**

We retrospectively identified all adults admitted to 5 Massachusetts hospitals from 2015–2022 with suspected sepsis or septic shock (blood cultures drawn and IV antibiotics within 24h of arrival, plus evidence of organ dysfunction for sepsis and hypotension or lactate ≥4.0mmol/L for septic shock). We used multivariable logistic regression to calculate associations between recorded race/ethnicity/sex and antibiotic receipt within 3–6h vs 0–3h, adjusting for language, other demographics, infection source, comorbidities, allergies, labs, and vitals. We then calculated associations between race/ethnicity/sex and in-hospital mortality with and without indicators for time-to-antibiotics.

**Results:**

The cohort included 48,263 patients with suspected sepsis or septic shock. Women had longer median time-to-antibiotics vs men for both sepsis (202 vs 188min; adjusted odds ratio [aOR] for 3–6h vs 0–3h, 1.15 [95% CI, 1.07–1.25]) and septic shock (159 vs 141min; aOR 1.09 [95% CI, 1.01–1.17]) (Table and Figure A). Among race/ethnicity categories, differences in time-to-antibiotics were most pronounced when comparing Black vs White patients (Figure A), which were significant for both sepsis (median 212 vs 193min; aOR for 3–6h vs 0–3h, 1.23 [95% CI, 1.06–1.43]) and septic shock (median 157 vs 147min; aOR 1.27 [95% CI, 1.08–1.48]). There was no association between race/ethnicity/sex and in-hospital mortality for sepsis without shock; however, women with septic shock had higher mortality (aOR 1.15 [95% CI, 1.05–1.27]). This difference persisted after adjusting for time-to-antibiotics (aOR 1.17 [95% CI, 1.03–1.32]) (Figure B).

**Table.**
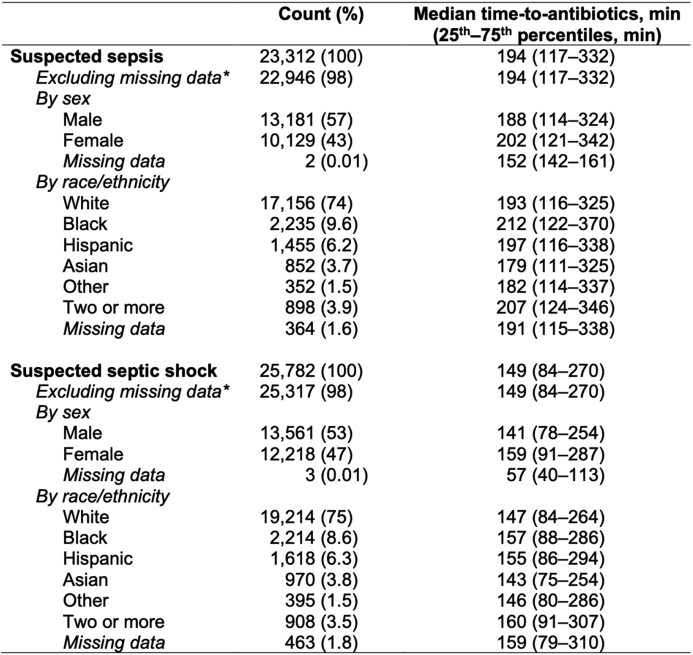
Cohort sizes and median time-to-antibiotics by race/ethnicity and sex. * Excluding the patients with either missing sex or missing race/ethnicity. These patients were not included in the multivariable regression models.

**Figure.**
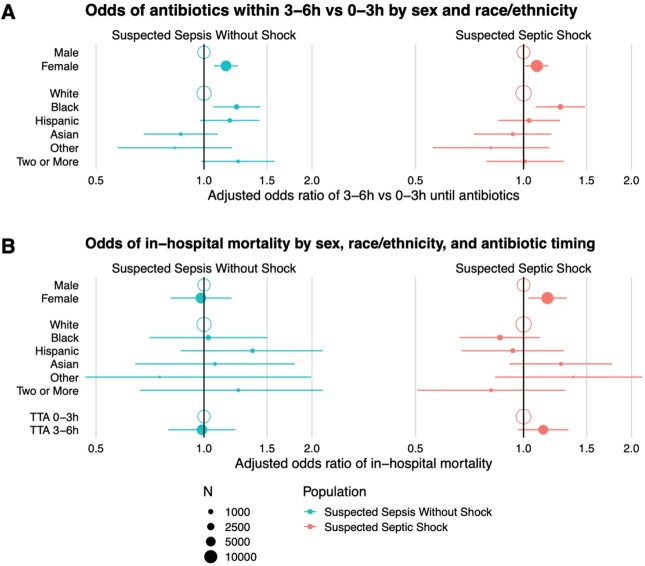
Disparities in time-to-antibiotics and in-hospital mortality by sex and race/ethnicity for sepsis with and without shock.

Multivariable regression was used to model associations between race/ethnicity/sex and odds of antibiotics within 3-6h vs 0-3h (A) or in-hospital mortality (B), adjusting for potential confounders (see Abstract text). In-hospital mortality was additionally adjusted for time-to-antibiotics (TTA) using the same categorization. Models included interaction terms between either race/ethnicity and sex (A) or all combinations of race/ethnicity, sex, and TTA (B), but none were significant at P<0.05, and they are not depicted in this figure. Adjusted odds ratios are drawn with 95% confidence intervals (horizontal lines). The X-axis is logarithmically scaled. Point sizes are scaled to the number of each patients in each subcohort (N, legend).

**Conclusion:**

In a large cohort of patients with sepsis, both women and Black patients had longer delays until antibiotics, which persisted after adjusting for multiple potential confounders. Women with septic shock also had greater in-hospital mortality, even when accounting for antibiotic delays.

**Disclosures:**

**Chanu Rhee, MD, MPH**, Cytovale: Advisor/Consultant|Pfizer: Advisor/Consultant|UpToDate, Inc.: Honoraria **Michael Klompas, MD, MPH**, UpToDate, Inc.: Royalties for chapters on pneumonia

